# Effectiveness of intrapartum azithromycin to prevent infections in planned vaginal births in low-income and middle-income countries: a post-hoc analysis of data from a multicentre, randomised, double-blind, placebo-controlled trial

**DOI:** 10.1016/S2214-109X(24)00562-X

**Published:** 2025-03-26

**Authors:** Waldemar A Carlo, Alan T N Tita, Janet L Moore, Musaku Mwenechanya, Elwyn Chomba, Jennifer J Hemingway-Foday, Avinash Kavi, Mrityunjay C Metgud, Shivaprasad S Goudar, Richard J Derman, Adrien Lokangaka, Antoinette Tshefu, Melissa Bauserman, Jackie K Patterson, Poonam Shivkumar, Manju Waikar, Archana Patel, Patricia L Hibberd, Paul Nyongesa, Fabian Esamai, Osayame Austine Ekhaguere, Sherri Bucher, Saleem Jessani, Shiyam Sunder Tikmani, Sarah Saleem, Robert L Goldenberg, Sk Masum Billah, Ruth Lennox, Rashidul Haque, William Petri, Manolo Mazariegos, Nancy F Krebs, Denise C Babineau, Elizabeth M McClure, Marion Koso-Thomas, A-PLUS Trial Group, Trecious Mweemba, Ernest Banda, Mwansa Chimfwembe, Ruth Nakazwe, Monica Collins, Sixto Leal, Akila Subramaniam, Charitharth Vivek Lal, Suchita Parepalli, Anna Aceituno, Jean Kim, Kay Jackson, Alexis Williams, Marissa Trotta, Menachem Miodovnik, Jackie Wallace, Gustave Lomendje, Michel Kalonji, Miyalu Junior, Jackie Patterson, Paulin Takoy, Justin Gado, Joel Bossenya, Emmanuel Kalombo, Charles Kombi, Maynor Manrique, Jamie Westcott, Shahjahan Siraj, Wazi Sadeq-ur-Rahman, Lolit Singh, Amita Farzana, Farhana Jahan, Zarin Tasmin Maliha, Rumpa Kairy, Christian Chisolm, Robert A Sinkin, Manjunath S Somannavar, Kadappa Beniwadi, Sheetal U Harkuni, Madiwalayya S. Ganarchari, Umesh R Hundekar, Ashadevi Patil, Ashwini Bharamashetti, Netravathi Alur, Surekha Nyamagoud, Chandrashekhar Kajagar, Sangappa M Dhaded, Ashalata A Mallapur, Ramesh Pol, Geetanjali M Katageri, Umesh Y Ramadurg, Bhuvaneshwari C Yelamali, Aarti Bhurle, Shailaja R Bidri, Sangamesh S Mathapati, Mallanagowda M Patil, Preeti G Patil, Hidayatullah R Bijapure, Chandrika R Doddihal, Muttu R Gudadinni, Smita O Bagali, Frances Jaeger, Farnaz Naqvi, Naija Karim Ghanchi, Zaheer Habib, Imran Ahmed, Sana Roujani, Seemab Naqvi, Sayyeda Reza, Haleema Yasmin, Mashal Khan, Mehmood Shaikh, Hayat Bozdar, Prabir Dad, Kunal G Kurhe, Vaishali Khedikar, Chaitali Gedam, Savita Bhargav, Samreen Sadaf, Deepti Shrivastava, Abhay Gaidhane, Mugdha Jungari, Manish Jain, Manisha Nasre, Sunanda Shrikhande, Vijayshri Deotale, Edwar A Liechty, Amos Sagwe, Kevin Otieno, Milsort Kemboi, Anderson Misati, Gabriel Kigen

**Affiliations:** a University of Alabama at Birmingham, Birmingham, AL, USA; b RTI International, Durham, NC, USA; c University Teaching Hospital, Lusaka, Zambia; d Women's and Children's Health Research Unit, KLE Academy of Higher Education and Research's JN Medical College, Belagavi, India; e Thomas Jefferson University, Philadelphia, PA, USA; f Kinshasa School of Public Health, Kinshasa, Democratic Republic of the Congo; g University of North Carolina at Chapel Hill, Chapel Hill, NC, USA; h Mahatma Gandhi Institute of Medical Sciences, Sevagram, India; i Government Medical College, Nagpur, India; j Lata Medical Research Foundation, Nagpur, India; k Datta Meghe Institute of Medical Sciences, Wardha, India; l Boston University School of Public Health, Boston, MA, USA; m Moi University School of Medicine, Eldoret, Kenya; n Indiana School of Medicine, University of Indiana, Indianapolis, IN, USA; o Aga Khan University, Karachi, Pakistan; p Columbia University School of Medicine, New York, NY, USA; q International Centre for Diarrhoeal Disease Research, Dhaka, Bangladesh; r University of Sydney, Sydney, NSW, Australia; s LAMB Hospital, Parbatipur, Bangladesh; t University of Virginia, Charlottesville, VA, USA; u Instituto de Nutrición de Centroamérica y Panamá, Guatemala City, Guatemala; v University of Colorado—Anschutz Medical Campus, Denver, CO, USA; w Eunice Kennedy Shriver National Institute of Child Health and Human Development, Bethesda, MD, USA

## Abstract

**Background:**

In 2023, the Azithromycin Prevention in Labor Use (A-PLUS) trial showed intrapartum azithromycin reduces maternal sepsis or death in women with planned vaginal delivery in low-resource settings, but whether it reduces maternal infection is unknown. We aimed to evaluate the effectiveness of intrapartum azithromycin in reducing maternal infection.

**Methods:**

We performed a post-hoc analysis of the multicentre, facility-based, randomised, double-blind, placebo-controlled A-PLUS trial. This trial compared prophylactic intrapartum single oral dose of 2 g azithromycin versus placebo on maternal morbidity and mortality in low-resource settings in southeast Asia and Africa from Sept 9, 2020, to Aug 18, 2022. The trial enrolled women in labour at 28 weeks' gestation (or later) at eight sites in the Democratic Republic of the Congo, Kenya, Zambia, Bangladesh, India, Pakistan, and Guatemala and found that azithromycin reduced the incidence of maternal sepsis or death. The primary outcome of the present analysis was the incidence of any maternal infection in the azithromycin versus placebo groups, which was defined as one or more of these infections after randomisation: chorioamnionitis, endometritis, perineal or caesarean wound infection, abdominopelvic abscess, mastitis or breast abscess, and other infections. Any neonatal infection was also analysed. All analyses were by intention to treat in all those with data available for that outcome. Relative risks (RRs) and 95% CIs were estimated with a Poisson model adjusted for treatment group and site. Subgroup analyses included a two-way interaction test between intervention group and subgroup. A-PLUS was registered at ClinicalTrials.gov, number NCT03871491.

**Findings:**

29 278 women were randomly assigned to groups: 14 590 to receive azithromycin, 14 688 to receive placebo. Baseline characteristics were similar between the azithromycin and placebo groups (43·3% vs 43·4% primiparous, 8·5% vs 8·7% high risk for infection). The presence of any maternal infection occurred less often in the azithromycin group (580 [4·0%] of 14 558) compared with the placebo group (824 [5·6%] of 14 661 women; RR 0·71, 95% CI 0·64–0·79, p<0·0001). Any neonatal infection did not differ between treatment groups. Adverse events were not detected.

**Interpretation:**

Among women planning vaginal delivery, this analysis provides evidence indicating that intrapartum azithromycin is associated with a lower incidence of maternal infections than placebo.

**Funding:**

The Eunice Kennedy Shriver National Institute of Child Health and Human Development and Bill and Melinda Gates Foundation via Foundation of National Institutes of Health.

**Translations:**

For the French and Spanish translations of the abstract see Supplementary Materials section.

## Introduction

Maternal infections occur in 5–8% of pregnancies,^1^, ^2^ accounting for approximately 11% of the global burden of maternal deaths, the third most common cause of maternal death worldwide.^3^ Neonatal infections are the third most common cause of neonatal mortality and account for about 16% of neonatal mortality worldwide.^4^ Severe infections that lead to life-threatening organ dysfunction are classified as sepsis. Maternal sepsis is defined by WHO as “the presence of suspected or confirmed infection plus signs of mild to moderate organ dysfunction (eg, tachycardia, low blood pressure, tachypnoea, altered mental status, reduced urinary output)”.^5^ Maternal and neonatal deaths from infections are not decreasing.^6^, ^7^ The available preventive or treatment strategies have had little effect, and WHO grades the evidence generally as low or very low quality.^8^ Data on appropriate perinatal antibiotic use are scarce, and many factors can determine how health-care providers use or abuse antiseptic and antibiotic treatments to prevent or treat maternal infections, according to a systematic review.^9^ Prophylaxis with antibiotics is recommended in selected clinical situations^8^ but it is not recommended to reduce infections in the vast majority of the deliveries. A 2024 systematic review and meta-analysis concluded that there is a need to develop local and international guidelines for the treatment of maternal infections.^10^

**Research in context**

**Evidence before this study**

Intrapartum azithromycin might reduce maternal and neonatal infections in low-resource settings but two of the large trials focused on sepsis and death rather than infections. The A-PLUS trial reported that intrapartum azithromycin reduces sepsis or death in low-income and middle-income countries (LMICs) but did not analyse maternal or neonatal infections. A 2024 meta-analysis concluded that intrapartum azithromycin administration reduces postpartum infections, but few types of infections were included. We searched PubMed on Dec 3, 2024 for manuscripts on maternal and neonatal infections after intrapartum azithromycin without date or language restriction using the search terms: “azithromycin” AND (“labor” OR “intrapartum” OR “pregnancy”) AND (“infection” OR “sepsis” OR “maternal mortality” OR “neonatal mortality”). Our search yielded 1041 articles, of which five were randomised controlled trials and four were meta-analyses that reported data from women with intended vaginal deliveries in LMICs.

**Added value of this study**

Our study showed that maternal infections of any type occurred less often in the azithromycin group compared with the placebo group, and that the effect was greater against any maternal infection than maternal sepsis or death. Intrapartum azithromycin was not associated with a lower rate of meeting accepted criteria for maternal sepsis if there was no suspicion of an additional infection.

**Implications of all the available evidence**

The reduction of maternal infections affirms and expands our understanding of the benefits of intrapartum azithromycin in planned vaginal deliveries beyond maternal sepsis. The expanded antimicrobial coverage provided by azithromycin might be particularly beneficial to prevent maternal infections, as adjunctive prophylactic azithromycin was also effective in reducing maternal infections in caesarean deliveries. Future research could assess the effectiveness of the implementation of intrapartum azithromycin in selected geographical or practice settings (eg, in Africa) or could test azithromycin against other frequently used antibiotics in settings of high antibiotic use. Ongoing studies are evaluating the association of the single dose with azithromycin resistance.

Intrapartum oral azithromycin prophylaxis (2 g) is a promising intervention that has been tested to reduce infections in four randomised controlled trials in low-income and middle-income countries (LMICs).^11^, ^12^, ^13^, ^14^ Two single-country pilot trials of intrapartum azithromycin conducted in The Gambia and Cameroon reported large reductions in any maternal^9^, ^11^ or any neonatal infection^9^ associated with this antibiotic, but the small number of participants in both trials precluded reliable estimates of infection rates, confidence in the results, and analysis of major outcomes including deaths. In the largest trial to date (Azithromycin Prevention in Labor Use Study [A-PLUS]),^13^ which included 29 278 women in seven countries, maternal sepsis or death was decreased in the azithromycin group compared with the placebo group (1·6% *vs* 2·4%; relative risk [RR] 0·67, 95% CI 0·56–0·79; number needed to treat 125), due to decreased incidence of sepsis. Although some individual types of maternal infections were decreased in the A-PLUS trial, a composite outcome of any type of maternal infection was not reported in the A-PLUS trial. By contrast, in the second largest trial of intrapartum azithromycin, which was conducted in The Gambia and Burkina Faso and enrolled 11 983 women, a composite outcome of maternal sepsis or death was not reduced and the outcome of any maternal infection was low in both groups (0·4% azithromycin *vs* 0·7% placebo).^14^ A composite of neonatal sepsis or death was not reduced in either of the trials, but the trials in The Gambia and Burkina Faso reported a reduction in any neonatal infection from 4·4% to 3·0% with the introduction of azithromycin.^13^, ^14^ A composite outcome that included any neonatal infection was not reported in the A-PLUS trial. A 2024 meta-analysis concluded that intrapartum azithromycin administration reduces postpartum infections but the type of infections included was limited.^15^ Given the importance of maternal and neonatal infections but the scarce data on the composite outcome of any infections in the intrapartum azithromycin trials, we aimed to perform a post-hoc analysis to test the hypothesis that a single oral dose of intrapartum azithromycin in women in labour planning a vaginal delivery was associated with a lower incidence of any maternal infection.

## Methods

### Trial design and participants

This study was a post-hoc analysis of the A-PLUS trial,^11^ a placebo-controlled randomised clinical trial of 2 g of azithromycin given orally to women in labour at 28 weeks or later of pregnancy in health facilities of the eight sites (Bangladesh, the Democratic Republic of the Congo, Guatemala, Kenya, two sites in India, Pakistan, and Zambia) of the Eunice Kennedy Shriver National Institute of Child Health and Human Development (NICHD) Global Network for Women's and Children's Health Research. Participants were assigned (1:1) by random allocation sequence using sequentially numbered envelopes and participants, care providers, research personnel, and outcome assessors were masked to the intervention group. All participants provided informed consent before enrolment. The full methods and procedures of the trial (including randomisation), the algorithm for maternal and neonatal sepsis, and the protocol have been previously reported.^11^, ^16^ An interim analysis that was conducted when 76% of participants were randomly assigned indicated maternal benefit; consequently, the trial was stopped for efficacy at the recommendation of the independent data monitoring committee. Participants in this post-hoc analysis were all participants who were included in the intention-to-treat population in the A-PLUS trial. The current post-hoc analysis was prespecified after the initial study was completed but before this analysis was conducted. The institutional review boards and ethics review committees at each participating Global Network for Women's and Children's Health Research site and their partner US institutions and the data coordinating centre approved the A-PLUS trial protocol.^11^ A steering committee and an independent data monitoring committee appointed by NICHD provided safety and monitoring of the trial. A-PLUS was registered at ClinicalTrials.gov, number NCT03871491.

### Outcomes

Any maternal infection was defined as one or more of the following: chorioamnionitis, endometritis, wound infection, abdominopelvic abscess, mastitis or breast abscess or infection, pyelonephritis, pneumonia, and other suspected infection documented in the clinical record (appendix 3 p 23). The WHO definition for maternal sepsis was operationalised as suspected or confirmed infection based on the presence of fever (>100·4°F or >38°C) or hypothermia (<96·8°F or <36°C) plus one or more signs of mild to moderate organ dysfunction including tachycardia (≥120 beats per min), low blood pressure (systolic <90 mm Hg), tachypnoea (>24 breaths per min), altered mental status or confusion, reduced urinary output (<500 mL over 24 h), jaundice, or renal failure (>1·2 mg/dL). The combinations of the presence or absence of maternal sepsis and any maternal infection were classified as sepsis with another infection, sepsis without other infection, and maternal infection without sepsis.

Any neonatal infection included neonatal sepsis and other infections. Neonatal sepsis was defined as proven (positive bacterial blood culture) or possible serious bacterial infection, pneumonia, or meningitis. Possible serious bacterial infection required one or more of the following WHO criteria: severe chest in-drawing, fever (≥100·4°F or ≥38·0°C), hypothermia (<95·9°F or <35·5°C), no movement or movement only on stimulation, poor or no feeding, or convulsions. Other neonatal infections included urinary infection, omphalitis, eye infection with swelling or drainage, skin infection with 10 or more pustules or bullae, pyelonephritis or kidney infection, other infection documented in the clinical record, or respiratory rate more than 60 breaths per min.

Infection was classified as any infection, sepsis plus another infection, sepsis without another infection, infection without sepsis, and a composite of maternal death, sepsis, or infection. In the infants, analyses compared the incidence of stillbirth, neonatal death between birth and 28 days postpartum, or at least one neonatal infection between birth and 28 days post-partum between treatment groups. Types of infection were neonatal sepsis, neonatal infection, and neonatal infection without sepsis. Any outcome involving infection was broken down also by type of infection. Subgroup analyses of each of these outcomes were also conducted in the following subgroups: high risk and low risk for infection (high risk if membrane ruptured ≥8 h or in labour ≥18 h before randomisation), region (Africa or Asia), prophylactic antibiotic, delivery mode, type of labour, and preterm and term status.

Safety monitoring of harms or unintended effects was based on surveillance of maternal side-effects (eg, nausea, vomiting, and diarrhoea, abdominal pain, vaginitis, and dizziness) potentially associated with azithromycin during labour and postpartum. For infants, findings suggestive of pyloric stenosis were assessed during the follow-up visits. Maternal and neonatal surveillance also included assessment of unintended medical visits and death from any cause. Additional maternal and neonatal risks monitored included anaphylaxis, allergic reactions, liver failure, and arrhythmias.

### Statistical methods

All analyses were performed in the intention-to-treat population, defined as all women who were randomly assigned and their infants, in all those with data available for each outcome. Baseline characteristics and outcomes in each treatment group were summarised with frequencies and percentages. The analyses were done to compare the incidence of at least one maternal infection diagnosed between delivery and 42 days postpartum (except chorioamnionitis, which is before delivery) between treatment groups. The RR and 95% CI of each outcome comparing the azithromycin group to the placebo group was obtained by fitting a generalised linear model with a Poisson distribution and log link and adjusting for site and treatment group as fixed effects. Subgroup analyses were performed by including fixed effects for the subgroup and a two-way interaction between treatment group and subgroup to find out if the effect of intrapartum azithromycin on the incidence of each outcome was modified by the subgroup.

Models for neonatal outcomes used a generalised estimating equation approach with a compound symmetry covariance structure to account for correlation of responses within multiple births. Due to the small number of participants that were lost to follow-up, the percentage of participants with missing outcomes was negligible across all outcomes (<0·5%) and so all analyses were performed by assuming any missing data were completely at random. All analyses were exploratory in nature and so no adjustment was made for multiple comparisons arising from analyses of multiple outcomes or subgroups and p values and CIs were provided for descriptive purposes only. All analyses were conducted with SAS version 9.4 and figures were produced using R version 4.4.0.

### Role of the funding source

The funders of the study had no role in the study design, data collection, data analysis, data interpretation, or writing of the report.

## Results

**Figure 1:**
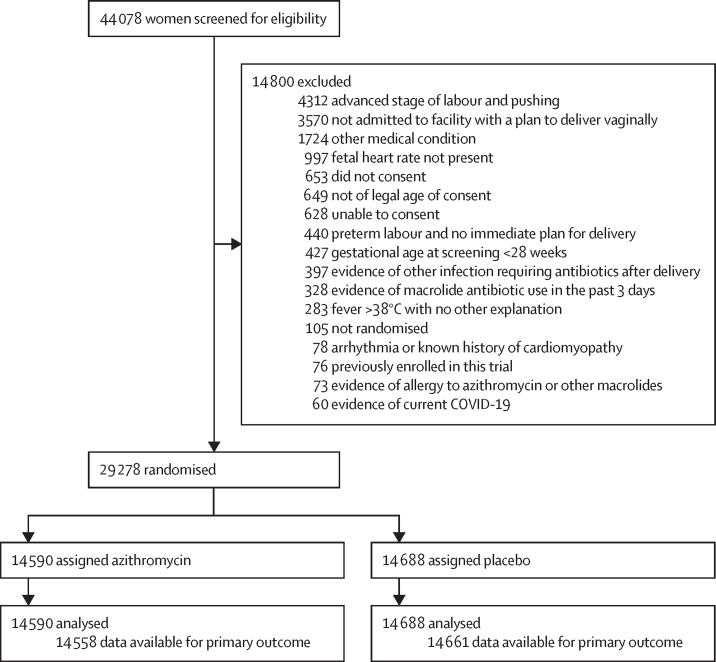
Participant flow diagram

29 278 participants were randomly assigned to either the azithromycin group (14 590 women with 14 687 neonates or stillbirths) or to the placebo group (14 688 women with 14 782 neonates or stillbirths) from Sept 9, 2020 up to Aug 18, 2022. Baseline characteristics of the two groups did not differ (table 1; figure 1). Maternal participants were followed up between 7 days and 7 months after birth and neonatal participants were followed up between 7 days and 8 months after birth.

**Table 1:** Baseline, labour, and delivery characteristics by treatment group

		**Azithromycin (n=14 590)**	**Placebo (n=14 688)**
Region			
	Africa	5779/14 590 (39·6%)	5801/14 688 (39·5%)
	Asia	8017/14 590 (54·9%)	8084/14 688 (55·0%)
	Latin America	794/14 590 (5·4%)	803/14 688 (5·5%)
Median maternal age (IQR), years		24·0 (21·0–28·0)	24·0 (21·0–28·0)
Married		13 729/14 589 (94·1%)	13 834/14 687 (94·2%)
Maternal education			
	No formal schooling	3457/14 565 (23·7%)	3476/14 665 (23·7%)
	1–6 years of schooling	2002/14 565 (13·7%)	2022/14 665 (13·8%)
	7–12 years of schooling	7308/14 565 (50·2%)	7325/14 665 (49·9%)
	≥13 years of schooling	1798/14 565 (12·3%)	1842/14 665 (12·6%)
Primiparous		6311/14 588 (43·3%)	6376/14 687 (43·4%)
Multiple birth		99/14 588 (0·7%)	95/14 687 (0·6%)
Any maternal infection during pregnancy*		797/14 589 (5·5%)	821/14 687 (5·6%)
Any maternal condition during pregnancy†		1017/14 589 (7·0%)	955/14 687 (6·5%)
Gestational age <37 weeks		1841/14 583 (12·6%)	1895/14 684 (12·9%)
Labour induction		2651/14 581 (18·2%)	2724/14 677 (18·6%)
High risk for sepsis before randomisation		1247/14 588 (8·5%)	1283/14 687 (8·7%)
Prolonged labour ≥18 h before randomisation		670/14 588 (4·6%)	698/14 687 (4·8%)
Prolonged rupture of membranes ≥8 h before randomisation		615/14 588 (4·2%)	632/14 687 (4·3%)

Data are n/N (%), unless otherwise specified.

* Any maternal infection during pregnancy includes group B streptococcus, pneumonia, pyelonephritis, rubella, chlamydia, herpes, syphilis, gonorrhoea, HIV, hepatitis B, malaria, urinary tract infection, or other infection.

† Any maternal condition during pregnancy includes diabetes, chronic hypertension, hypertensive disorders of pregnancy, or other condition.

Any maternal infection occurred less often in the azithromycin group compared with the placebo group (4·0% *vs* 5·6%; RR 0·71, 95% CI 0·64–0·79; p<0·0001; table 2). Sepsis plus another infection and any infection without sepsis occurred less often in the azithromycin group. By contrast, there was no evidence of a statistically significance difference in the incidence of sepsis without another infection between groups. The results for the specific type of infections are in table 2. The composite outcome of any maternal infection or death occurred less often in the azithromycin group compared with the placebo group (4·0% *vs* 5·7%; RR 0·72, 95% CI 0·64–0·80; table 2).

**Table 2:** Maternal and newborn outcomes by treatment

				**Azithromycin**	**Placebo**	**Relative risk (95% CI)**
**Maternal outcome**						
Any infection				580/14 558 (4·0%)	824/14 661 (5·6%)	0·71 (0·64–0·79)
	Sepsis without another infection			69/14 558 (0·5%)	76/14 658 (0·5%)	0·91 (0·66–1·26)
	Sepsis plus another infection			150/14 558 (1·0%)	261/14 658 (1·8%)	0·58 (0·47–0·71)
		Chorioamnionitis		1/14 558 (<0·1%)	3/14 662 (<0·1%)	..
		Endometritis		118/14 558 (0·8%)	205/14 659 (1·4%)	0·58 (0·46–0·73)
		Wound infection		24/14 557 (0·2%)	46/14 655 (0·3%)	0·53 (0·32–0·86)
		Other maternal infection		56/14 558 (0·4%)	79/14 657 (0·5%)	0·71 (0·51–1·01)
			Abdominopelvic abscess	2/14 558 (<0·1%)	3/14 657 (<0·1%)	0·67 (0·11–4·01)
			Mastitis or breast abscess or infection	21/14 558 (0·1%)	27/14 655 (0·2%)	0·78 (0·44–1·38)
			Pyelonephritis	5/14 558 (<0·1%)	23/14 655 (0·2%)	0·22 (0·08–0·58)
			Pneumonia	29/14 558 (0·2%)	27/14 657 (0·2%)	1·09 (0·64–1·83)
	Infection without sepsis			361/14 558 (2·5%)	485/14 658 (3·3%)	0·75 (0·66–0·86)
		Chorioamnionitis		4/14 558 (<0·1%)	5/14 662 (<0·1%)	..
		Endometritis		73/14 558 (0·5%)	89/14 659 (0·6%)	0·83 (0·61–1·13)
		Wound infection		202/14 557 (1·4%)	276/14 655 (1·9%)	0·74 (0·61–0·88)
		Other maternal infection		93/14 558 (0·6%)	138/14 657 (0·9%)	0·68 (0·52–0·89)
			Abdominopelvic abscess	2/14 558 (<0·1%)	3/14 657 (<0·1%)	..
			Mastitis or breast abscess or infection	17/14 558 (0·1%)	30/14 655 (0·2%)	0·57 (0·32–1·04)
			Pyelonephritis	6/14 558 (<0·1%)	20/14 655 (0·1%)	0·30 (0·12–0·76)
			Pneumonia	0/14 558	4/14 657 (<0·1%)	..
			Other bacterial infection	68/14 558 (0·5%)	81/14 657 (0·6%)	0·85 (0·61–1·17)
Death or infection				588/14 526 (4·0%)	829/14 640 (5·7%)	0·72 (0·64–0·80)
**Newborn outcome**						
Any infection				2196/14 573 (15·1%)	2205/14 657 (15·0%)	1·01 (0·96–1·06)
Infection without sepsis				763/14 573 (5·2%)	798/14 657 (5·4%)	0·97 (0·88–1·07)
	Eye infection with swelling or drainage			152/14 573 (1·0%)	124/14 657 (0·8%)	1·23 (0·98–1·56)
	Skin infection with ≥10 pustules or bullae			61/14 573 (0·4%)	92/14 657 (0·6%)	0·67 (0·49–0·93)
	Omphalitis			29/14 573 (0·2%)	36/14 657 (0·2%)	0·81 (0·50–1·32)
	Urinary tract infection			1/14 573 (<0·1%)	5/14 657 (<0·1%)	0·20 (0·02–1·72)
	Pyelonephritis or kidney infection			0/14 573	0/14 657	..
	Pneumonia or lung infection			15/14 573 (0·1%)	11/14 657 (0·1%)	1·37 (0·63–2·99)
	Meningitis			0/14 573	0/14 657	..
	Other infection in clinical record			128/14 573 (0·9%)	140/14 657 (1·0%)	0·92 (0·72–1·16)
	Respiratory rate ≥60 breaths per min			435/14 573 (3·0%)	448/14 657 (3·1%)	0·99 (0·87–1·12)
Stillbirth, death, or infection				2300/14 658 (15·7%)	2319/14 757 (15·7%)	1·00 (0·95–1·06)

Data are n/N (%), unless otherwise specified. Estimates were obtained by fitting a Poisson model to each outcome adjusting for site and treatment. Models for neonatal outcomes account for correlation among multiples assuming an exchangeable covariance structure. Models for skin infection, urinary tract infection, and pneumonia had convergence issues, so models are fit without the adjustment for correlation among multiples. Models for chorioamnionitis, pyelonephritis or kidney infection, and meningitis did not converge.

There was no evidence of a statistically significant difference between groups with respect to the incidence of any neonatal infection, any neonatal infection without sepsis, or the composite outcome of any infection, stillbirth, or neonatal death (table 2). Skin infections with ten or more pustules or bullae were lower in the azithromycin group than in the placebo group (0·4% *vs* 0·6%; RR 0·67, 95% CI 0·49–0·93). There was also no evidence of a statistically significant difference between groups in the incidence of other neonatal infections (table 2).

**Figure 2:**
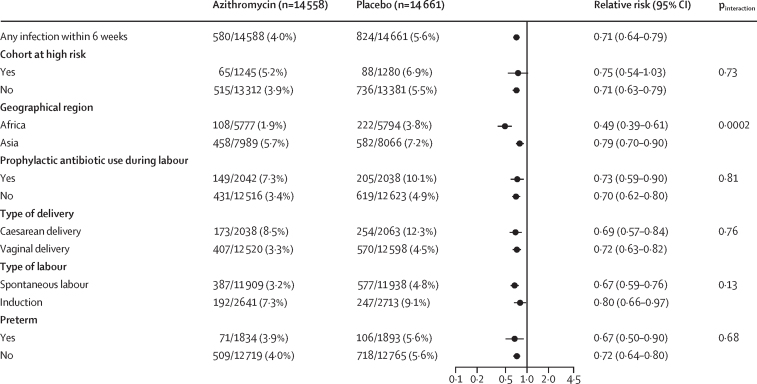
Any maternal infection subgroup analyses Forest plot displaying the estimated relative risk (95% CI) of maternal sepsis or infection comparing mothers randomly assigned to azithromycin versus placebo overall and within selected subgroups. Overall estimates were obtained by fitting a Poisson model adjusting for site and treatment. Subgroup estimates were obtained by fitting a Poisson model adjusting for site, treatment, subgroup, and the interaction of treatment and subgroup.

**Figure 3:**
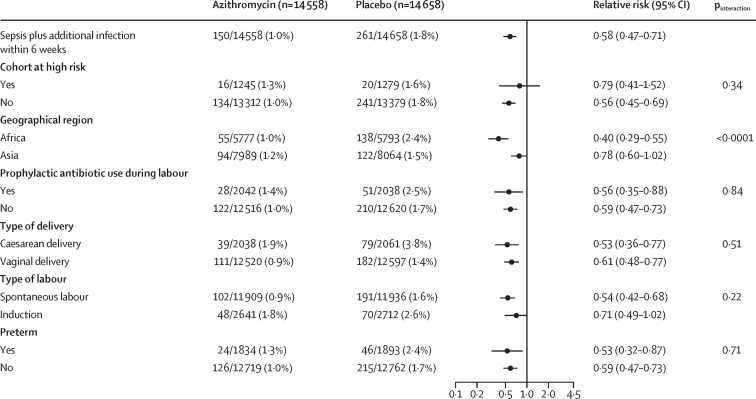
Maternal sepsis plus another infection subgroup analyses Forest plot displaying the estimated relative risk (95% CI) of maternal sepsis with infection comparing mothers randomly assigned to azithromycin versus placebo overall and within selected subgroups. Overall estimates were obtained by fitting a Poisson model adjusting for site and treatment. Subgroup estimates were obtained by fitting a Poisson model adjusting for site, treatment, subgroup, and the interaction of treatment and subgroup.

**Figure 4:**
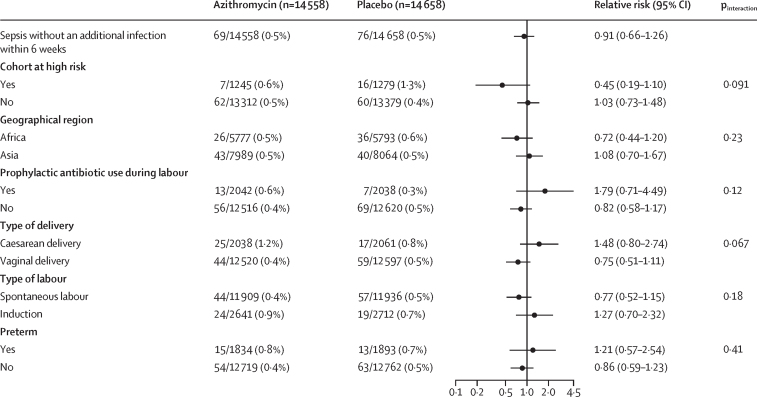
Maternal sepsis without an additional infection subgroup analyses Forest plot displaying the estimated relative risk (95% CI) of maternal sepsis without infection comparing mothers randomly assigned to azithromycin versus placebo overall and within selected subgroups. Overall estimates were obtained by fitting a Poisson model adjusting for site and treatment. Subgroup estimates were obtained by fitting a Poisson model adjusting for site, treatment, subgroup, and the interaction of treatment and subgroup.

In the prespecified subgroup analyses of maternal outcomes, the effect of azithromycin on reducing the incidence of any maternal infection was stronger in Africa than Asia (p<0·0001; figure 2), maternal sepsis plus another infection (p<0·0001; figure 3), and any maternal infection or death (p<0·0001; appendix 3 p 9). There was no evidence that geographical region modified the effect of azithromycin on the incidence of maternal sepsis without another infection (p=0·23; figure 4) or maternal infection without sepsis (p=0·21; appendix 3 p 9). There was also no evidence that the cohort at high risk, prophylactic antibiotic use during labour, type of delivery (caesarean or vaginal), type of labour (ie, spontaneous labour or induction), or preterm delivery modified the effect of azithromycin on any of the maternal outcomes.

In the subgroup analyses for the neonatal outcomes, including any infection, infection without sepsis, and the composite outcome of any infection, stillbirth, or neonatal death, there was no evidence of effect modification by any of the subgroups considered (appendix 3 pp 10–12). There were no harms or unintended effects reported in the A-PLUS Trial.^11^

## Discussion

In this post-hoc analysis of the A-PLUS trial, a single dose of intrapartum azithromycin was associated with a lower incidence of any maternal infection than placebo regardless of whether the maternal infection was associated with sepsis or not, without evidence of harm. This post-hoc analysis supports the results of the primary publication of the A-PLUS Trial^13^ and also shows that the analysis of a composite of infections, including infections not reported in the original manuscript from the randomised trial, showed a larger reduction compared with the reduction in sepsis previously reported.^13^ Furthermore, this analysis also found that, in the absence of another identified or suspected infection, azithromycin was not associated with a lower incidence of maternal sepsis, as defined by WHO, compared with placebo. These findings support the likelihood that the mechanism of action of intrapartum azithromycin is an antibiotic-mediated effect, as the positive associations were linked more to maternal infection than maternal sepsis, which was sometimes not associated with proven or suspected infection. The previous reports of azithromycin trials^12^, ^14^, ^16^ and a new meta-analysis^15^ did not report a composite of any maternal or any neonatal infection so this study reports unique data.

Although this post-hoc analysis was done strictly by randomisation with data from the large placebo-controlled A-PLUS trial, there are important limitations. First, this analysis was not prespecified in the original protocol, it was proposed after the data on death, sepsis, and some individual infections were analysed but before data on all maternal and neonatal infection were analysed. Still, as a post-hoc analysis of a randomised controlled trial, the best interpretation of the results is of associations rather than causal inferences. Thus, the results can only be considered exploratory. A second important limitation is that the accurate identification of maternal and neonatal infection is difficult, especially in low-resource settings, where incomplete laboratory support is available, and diagnoses are mostly dependent on clinical assessments. In preparation for the A-PLUS trial, capacity was built for improved clinical diagnosis and laboratory testing. Urinalysis and urine cultures were rarely collected, and urinary tract infections were rarely diagnosed. However, the inclusion of data on other maternal infections besides peripartum infections is an important strength of this study as maternal infections, other than peripartum infections, are common and account for many peripartum hospitalisations and deaths.^17^ Furthermore, follow-up was done to 42 days postpartum, which is not available in many studies and databases. Importantly, receipt of antibiotics was not solely sufficient to meet criteria for infection. Third, we made no allowance for multiplicity.

The incidence of maternal peripartum infection can vary by risk factors, definitions, and capacity for diagnosis including clinical or laboratory criteria. In the only meta-analysis of peripartum infection, which was published in 2019 and included 111 studies from 46 countries, 3·9% (95% CI 1·8–6·8) of women had chorioamnionitis, 1·6% (0·9–2·5) had endometritis, 1·2% (1·0–1·5) had wound infection, 0·05% (0·03–0·07) had sepsis, and 1·1% (0·3–2·4) had two or more maternal peripartum infections, for a total incidence of 7·8% women having one or more peripartum infections.^1^ Despite the limited data from LMICs in the meta-analysis, the incidence of maternal peripartum infection was similar to the data from the WHO-led multi-country (n=52) cross-sectional prospective study published in 2020, in which 7·0% of all women, 7·1% of women participating in low-income countries, and 7·2% of women living in lower-middle-income countries who gave birth were diagnosed with infection.^18^ The incidence of any maternal infection in the placebo group of the A-PLUS trial was 5·6%, which was similar to the rates reported (excluding endometriosis and sepsis, as they were excluded in our trial) in the meta-analysis (6·2%) and the cross sectional study (5·9%).^1^, ^17^ The incidence of any maternal infection was also similar to the model-based data from the 2017 Global Burden of Diseases, Injuries, and Risk Factors Study.^19^

In preparation for the A-PLUS trial, capacity building was conducted at each site before starting the trial. Capacity building included training in recognition of peripartum and neonatal infections and obtaining the appropriate laboratory testing, but did not include infection prevention or control. Capacity building probably improved the diagnosis of maternal and neonatal infections. Intrapartum screening and enrolment excluded women with chorioamnionitis and other infections at the time of consent and randomisation, explaining the lower overall peripartum infection in this study compared with the meta-analysis data. In this study, the incidence of other maternal infections besides chorioamnionitis was similar to or higher than that in the meta-analysis, suggesting accurate identification of maternal peripartum infection in the A-PLUS trial. Blood, urine, or other cultures and pathogen identification were obtained from 3·1% of the mothers and 28·7% of the neonates as reported,^13^ which is similar to the rates of infections diagnosed, but uptake was not universal if an infection was suspected.

The intrapartum azithromycin trials reported a wide range of maternal and neonatal infection and sepsis rates. In the initial pilot trial in The Gambia, the incidence of maternal infection (9·2%) and neonatal infection (23·8%) were very high in the control group and decreased significantly in the azithromycin group for both maternal (3·6%) and neonatal (18·1%) infections.^11^ The Cameroon trial did not report any maternal or any neonatal infection but reported a reduction in chorioamnionitis (3·2% to 1·2%) and wound infections (4·0% to 0·8%).^12^ The trial in The Gambia and Burkina Faso found a low incidence of any maternal infection, with a small reduction in the azithromycin group compared with placebo (0·4% *vs* 0·7%).^14^ The incidences of maternal and neonatal infections can vary widely depending on baseline risk factors, definitions, and diagnostic capacity.^17^ Despite different baseline incidences of infection in the four randomised controlled trials in LMICs, the findings of lower incidence of any maternal infection in the azithromycin trials could be robust, generalisable, and clinically important. Azithromycin is effective in reducing maternal infections after caesarean delivery^13^, ^20^, ^21^, ^22^ and is recommended for prophylaxis in caesarean delivery during labour.^23^ The spectrum of antimicrobial coverage provided by azithromycin might be particularly beneficial to prevent perinatal infections.^24^ However, the widespread use of intrapartum antibiotics could lead to antibiotic resistance, a potential threat that makes infections hard to treat and resistant organisms more likely to spread, so the balance between benefits and harms needs to be considered.

In summary, this post-hoc analysis of the A-PLUS trial shows that use of azithromycin in low-resource settings was associated with a lower incidence of any maternal infection but not a difference in any neonatal infections compared with placebo. These data might be useful for epidemiological, programmatic, and research endeavours, as maternal infections are an important cause of maternal mortality and morbidities.^6^, ^17^, ^25^

### Contributors

### Equitable partnership declaration

The authors of this paper have submitted an equitable partnership declaration (appendix 4). This statement allows researchers to describe how their work engages with researchers, communities, and environments in the countries of study. This statement is part of *The Lancet Global Health*'s broader goal to decolonise global health.

### Data sharing

De-identified participant data from the A-PLUS trial are available at the National Institute of Child Health and Human Development data repository (N-DASH): https://dash.nichd.nih.gov/. Data sharing is accessed and governed according to the procedures and policies of N-DASH.

## Declaration of interests

WAC has received support from the Eunice Kennedy Shriver National Institute of Child Health and Human Development (NICHD) and the Bill & Melinda Gates Foundation via Foundation of National Institutes of Health (NIH) for this study and manuscript. WAC has received grants from the Thrasher Research Fund, the National Health, Lung, and Blood Institute, and the American Heart Association. WAC has also participated in Enhancing Quality and Access to Achieve Equitable Maternal and Infant Health, Enhanced glucose Monitoring to improve Pregnancy Outcomes for Women Requiring medications for gestational diabetes, National Institute of Arthritis and Musculoskeletal and Skin Diseases Salmon Study, and Increased Milk Protein to Accrue Critical Tissue. ATNT received support from the NIH, NICHD, and the Bill & Melinda Gates Foundation via Foundation of NIH for this study; additionally, he has received grants from Mirvie and American Heart Association. All authors received support for this manuscript from the NICHD and Foundation for the NIH.

## Supplementary Materials

French translation of the abstract

Spanish translation of the abstract

Supplementary appendix 3

Equitable Partnership Declaration
