# Supplementary material for: Effectiveness of intrapartum azithromycin to prevent infections in planned vaginal births in low-income and middle-income countries: a post-hoc analysis of data from a multicentre, randomised, double-blind, placebo-controlled trial
Source: Lancet Glob Health. 2025 Mar 26;13(4):e689–97. doi: 10.1016/S2214-109X(24)00562-X (PMC11950427; doi:10.1016/S2214-109X(24)00562-X)
Supplement: French translation of the abstract [file mmc1.pdf]

THE LANCET
Global Health

Supplementary appendix 1

This translation in French was submitted by the authors and we reproduce it as supplied. It has not been peer reviewed. *The Lancet*'s editorial processes have only been applied to the original in English, which should serve as reference for this manuscript.

Cette traduction en français a été proposée par les auteurs et nous l'avons reproduite telle quelle. Elle n'a pas été examinée par des pairs. Les processus éditoriaux du *Lancet* n'ont été appliqués qu'à l'original en anglais et c'est cette version qui doit servir de référence pour ce manuscrit.

Supplement to: Carlo WA, Tita ATN, Moore JL, et al. Effectiveness of intrapartum azithromycin to prevent infections in planned vaginal births in low-income and middle-income countries: a post-hoc analysis of data from a multicentre, randomised, double-blind, placebo-controlled trial. *Lancet Glob Health* 2025; **13:** e689–97.

Résumé

Contexte

En 2023, l'essai A-PLUS *(Azithromycin Prevention in Labor Use)* a démontré que l'azithromycine intrapartum réduit le sepsis ou le décès maternel chez les femmes ayant prévu un accouchement vaginal dans des milieux à faibles ressources, mais on ne sait pas si elle réduit l'infection maternelle. Nous avons cherché à évaluer l'efficacité de l'azithromycine intrapartum dans la réduction de l'infection maternelle.

Méthodes

Nous avons effectué une analyse post-hoc de l'étude A-PLUS, un essai multicentrique, randomisé, en double aveugle et contrôlé par placebo. Cet essai a comparé une dose orale unique prophylactique intrapartum de 2 g d'azithromycine à un placebo sur la morbidité et la mortalité maternelles dans des milieux à faibles ressources en Asie du Sud-Est et en Afrique du 9 septembre 2020 au 18 août 2022. L'essai a enrôlé des femmes en travail à 28 semaines de gestation (ou plus tard) sur huit sites en République démocratique du Congo, au Kenya, en Zambie, au Bangladesh, en Inde, au Pakistan et au Guatemala et a constaté que l'azithromycine réduisait l'incidence de sepsis ou de décès maternel. L'issue principale de la présente analyse était l'incidence de toute infection maternelle dans les groupes azithromycine et placebo, qui était définie comme une ou plusieurs de ces infections après randomisation : chorioamniotite, endométrite, infection de plaie périnéale ou césarienne, abcès abdominopelvien, mastite ou abcès du sein et autres infections. Toute infection néonatale a également été analysée. Toutes les analyses ont été effectuées en intention de traiter chez toutes les personnes pour lesquelles des données étaient disponibles pour cette issue. Les risques relatifs (RR) et les IC à 95 % ont été estimés à l'aide d'un modèle de Poisson ajusté pour le groupe de traitement et le site. Les analyses de sous-groupes comprenaient un test d'interaction bidirectionnel entre le groupe d'intervention et le sous-groupe. A-PLUS a été enregistré sur ClinicalTrials.gov, numéro NCT03871491.

Résultats

29 278 femmes ont été réparties aléatoirement en groupes : 14 590 pour recevoir de l'azithromycine, 14 688 pour recevoir un placebo. Les caractéristiques initiales étaient similaires entre les groupes azithromycine et placebo (43,3 % contre 43,4 % de primipares, 8,5 % contre 8,7 % de risque élevé d'infection). La présence d'une infection maternelle était moins fréquente dans le groupe azithromycine (580 [4,0 %] sur 14 558) que dans le groupe placebo (824 [5,6 %] sur 14 661 femmes ; RR 0,71, IC à 95 % 0,64–0,79, p < 0,0001). L'infection néonatale ne différait pas entre les groupes de traitement. Aucun événement indésirable n'a été détecté.

Interprétation

Parmi les femmes qui prévoient un accouchement vaginal, cette analyse fournit des preuves indiquant que l'azithromycine intrapartum est associée à une incidence plus faible d'infections maternelles que le placebo.
