# Supplementary material for: Effectiveness of intrapartum azithromycin to prevent infections in planned vaginal births in low-income and middle-income countries: a post-hoc analysis of data from a multicentre, randomised, double-blind, placebo-controlled trial
Source: Lancet Glob Health. 2025 Mar 26;13(4):e689–97. doi: 10.1016/S2214-109X(24)00562-X (PMC11950427; doi:10.1016/S2214-109X(24)00562-X)
Supplement: Spanish translation of the abstract [file mmc2.pdf]

THE LANCET
Global Health

Supplementary appendix 2

This translation in Spanish was submitted by the authors and we reproduce it as supplied. It has not been peer reviewed. *The Lancet*'s editorial processes have only been applied to the original in English, which should serve as reference for this manuscript.

Los autores nos proporcionaron esta traducción al español y la reproducimos tal como nos fue entregada. No la hemos revisado. Los procesos editoriales de *The Lancet* se han aplicado únicamente al original en inglés, que debe servir de referencia para este manuscrito.

Supplement to: Carlo WA, Tita ATN, Moore JL, et al. Effectiveness of intrapartum azithromycin to prevent infections in planned vaginal births in low-income and middle-income countries: a post-hoc analysis of data from a multicentre, randomised, double-blind, placebo-controlled trial. *Lancet Glob Health* 2025; **13:** e689–97.

Resumen

Antecedentes

En 2023, el ensayo Azitromicina para la prevención del parto (A-PLUS) mostró que la azitromicina intraparto reduce la sepsis o la muerte materna en mujeres con parto vaginal planificado en entornos de bajos recursos, pero se desconoce si reduce la infección materna. Nuestro objetivo fue evaluar la eficacia de la azitromicina intraparto para reducir la infección materna.

Métodos

Realizamos un análisis post-hoc del ensayo A-PLUS, un ensayo multicéntrico, aleatorizado, doble ciego y controlado con placebo. Este ensayo comparó la administración intraparto de una dosis oral única profiláctica de 2 g de azitromicina frente a placebo en la morbilidad y la mortalidad maternas en entornos de bajos recursos en el sudeste asiático y África desde el 9 de septiembre de 2020 hasta el 18 de agosto de 2022. El ensayo inscribió a mujeres en trabajo de parto a las 28 semanas de gestación (o más tarde) en ocho sitios en la República Democrática del Congo, Kenia, Zambia, Bangladesh, India, Pakistán y Guatemala y encontró que la azitromicina redujo la incidencia de sepsis o muerte materna. El resultado primario del análisis presente fue la incidencia de cualquier infección materna en los grupos de azitromicina frente a placebo, que se definió como una o más de estas infecciones después de la aleatorización: corioamnionitis, endometritis, infección de la herida perineal o de cesárea, absceso abdominopélvico, mastitis o absceso mamario y otras infecciones. También se analizó cualquier infección neonatal. Todos los análisis se realizaron por intención de tratar en todos aquellos con datos disponibles para ese resultado. Los riesgos relativos (RR) y los IC del 95 % se calcularon con un modelo de Poisson ajustado para el grupo de tratamiento y el sitio. Los análisis de subgrupos incluyeron una prueba de interacción bidireccional entre el grupo de intervención y el subgrupo. A-PLUS se registró en ClinicalTrials.gov, número NCT03871491.

Hallazgos

Se asignaron aleatoriamente 29278 mujeres a los siguientes grupos: 14590 para recibir azitromicina, 14 688 para recibir placebo. Las características iniciales fueron similares entre los grupos de azitromicina y placebo (43,3 % frente a 43,4 % primíparas, 8,5 % frente a 8,7 % con alto riesgo de infección). La presencia de cualquier infección materna se produjo con menor frecuencia en el grupo de azitromicina (580 [4,0%] de 14.558) en comparación con el grupo placebo (824 [5,6%] de 14661 mujeres; RR 0,71, IC del 95% 0,64–0,79, p<0,0001). No hubo diferencias en la infección neonatal entre los grupos de tratamiento. No se detectaron eventos adversos.

Interpretación

Entre las mujeres que planean un parto vaginal, este análisis proporciona evidencia que indica que la azitromicina intraparto se asocia con una menor incidencia de infecciones maternas que el placebo.
