# Supplementary material for: Effectiveness of intrapartum azithromycin to prevent infections in planned vaginal births in low-income and middle-income countries: a post-hoc analysis of data from a multicentre, randomised, double-blind, placebo-controlled trial
Source: Lancet Glob Health. 2025 Mar 26;13(4):e689–97. doi: 10.1016/S2214-109X(24)00562-X (PMC11950427; doi:10.1016/S2214-109X(24)00562-X)
Supplement: Supplementary appendix 3 [file mmc3.pdf]

THE LANCET
Global Health

Supplementary appendix 3

This appendix formed part of the original submission and has been peer reviewed. We post it as supplied by the authors.

Supplement to: Carlo WA, Tita ATN, Moore JL, et al. Effectiveness of intrapartum azithromycin to prevent infections in planned vaginal births in low-income and middle-income countries: a post-hoc analysis of data from a multicentre, randomised, double-blind, placebo-controlled trial. *Lancet Glob Health* 2025; **13:** e689–97.

Table 1. Baseline, Labor, and Delivery Characteristics by Treatment

	Azithromycin (n=14,590)	Placebo (n=14,688)
Region, n (%)		
Africa	5,779 (39.6)	5,801 (39.5)
Asia	8,017 (54.9)	8,084 (55.0)
Latin America	794 (5.4)	803 (5.5)
Maternal age (years), Median (IQ range)	24.0 (21.0, 28.0)	24.0 (21.0, 28.0)
Married, n/N (%)	13,729/14,589 (94.1)	13,834/14,687 (94.2)
Maternal education, n (%)	14,565	14,665
No formal schooling	3,457 (23.7)	3,476 (23.7)
1 - 6 years of schooling	2,002 (13.7)	2,022 (13.8)
7 - 12 years of schooling	7,308 (50.2)	7,325 (49.9)
≥ 13 years of schooling	1,798 (12.3)	1,842 (12.6)
Primiparous, n/N (%)	6,311/14,588 (43.3)	6,376/14,687 (43.4)
Multiple birth, n/N (%)	99/14,588 (0.7)	95/14,687 (0.6)
Any maternal infection during pregnancy[1], n/N (%)	797/14,589 (5.5)	821/14,687 (5.6)
Any maternal condition during pregnancy[2], n/N (%)	1,017/14,589 (7.0)	955/14,687 (6.5)
Gestational age < 37 weeks, n/N (%)	1,841/14,583 (12.6)	1,895/14,684 (12.9)
Labor induction, n/N (%)	2,651/14,581 (18.2)	2,724/14,677 (18.6)
High-risk for sepsis before randomization, n/N (%)	1,247/14,588 (8.5)	1,283/14,687 (8.7)
Prolonged labor ≥ 18 hours before randomization	670/14,588 (4.6)	698/14,687 (4.8)
Prolonged ROM ≥ 8 hours before randomization	615/14,588 (4.2)	632/14,687 (4.3)

[1] Any maternal infection during pregnancy includes group B streptococcus, pneumonia, pyelonephritis, rubella, chlamydia, herpes, syphilis, gonorrhea, HIV, hepatitis B, malaria, urinary tract infection, or other infection.

[2] Any maternal condition during pregnancy includes diabetes, chronic hypertension, hypertensive disorders of pregnancy, or other condition.

Table 2. Maternal and Newborn Outcomes by Treatment

	Azithromycin	Placebo	RR (95% CI)[4]
**Maternal Outcome**			
Any infection[1]	580/14,558 (4.0)	824/14,661 (5.6)	0.71 (0.64, 0.79)
Sepsis without another infection	69/14,558 (0.5)	76/14,658 (0.5)	0.91 (0.66, 1.26)
Sepsis plus another infection	150/14,558 (1.0)	261/14,658 (1.8)	0.58 (0.47, 0.71)
Chorioamnionitis	1/14,558 (0.0)	3/14,662 (0.0)	--
Endometritis	118/14,558 (0.8)	205/14,659 (1.4)	0.58 (0.46, 0.73)
Wound infection	24/14,557 (0.2)	46/14,655 (0.3)	0.53 (0.32, 0.86)
Other maternal infection	56/14,558 (0.4)	79/14,657 (0.5)	0.71 (0.51, 1.01)
Abdominopelvic abscess	2/14,558 (0.0)	3/14,657 (0.0)	0.67 (0.11, 4.01)
Mastitis/breast abscess or infection	21/14,558 (0.1)	27/14,655 (0.2)	0.78 (0.44, 1.38)
Pyelonephritis	5/14,558 (0.0)	23/14,655 (0.2)	0.22 (0.08, 0.58)
Pneumonia	29/14,558 (0.2)	27/14,657 (0.2)	1.09 (0.64, 1.83)
Infection without sepsis	361/14,558 (2.5)	485/14,658 (3.3)	0.75 (0.66, 0.86)
Chorioamnionitis	4/14,558 (0.0)	5/14,662 (0.0)	--
Endometritis	73/14,558 (0.5)	89/14,659 (0.6)	0.83 (0.61, 1.13)
Wound infection	202/14,557 (1.4)	276/14,655 (1.9)	0.74 (0.61, 0.88)
Other maternal infection	93/14,558 (0.6)	138/14,657 (0.9)	0.68 (0.52, 0.89)
Abdominopelvic abscess	2/14,558 (0.0)	3/14,657 (0.0)	--
Mastitis/breast abscess or infection	17/14,558 (0.1)	30/14,655 (0.2)	0.57 (0.32, 1.04)
Pyelonephritis	6/14,558 (0.0)	20/14,655 (0.1)	0.30 (0.12, 0.76)
Pneumonia	0/14,558 (0.0)	4/14,657 (0.0)	--
Other bacterial infection[2]	68/14,558 (0.5)	81/14,657 (0.6)	0.85 (0.61, 1.17)
Death or infection	588/14,526 (4.0)	829/14,640 (5.7)	0.72 (0.64, 0.80)
**Newborn Outcome**			
Any infection[3]	2,196/14,573 (15.1)	2,205/14,657 (15.0)	1.01 (0.96, 1.06)
Infection without sepsis	763/14,573 (5.2)	798/14,657 (5.4)	0.97 (0.88, 1.07)
Eye infection with swelling/drainage	152/14,573 (1.0)	124/14,657 (0.8)	1.23 (0.98, 1.56)
Skin infection with 10+ pustules/bullae	61/14,573 (0.4)	92/14,657 (0.6)	0.67 (0.49, 0.93)
Omphalitis	29/14,573 (0.2)	36/14,657 (0.2)	0.81 (0.50, 1.32)
Urinary tract infection	1/14,573 (0.0)	5/14,657 (0.0)	0.20 (0.02, 1.72)
Pyelonephritis/Kidney infection	0/14,573 (0.0)	0/14,657 (0.0)	--
Pneumonia/Lung infection	15/14,573 (0.1)	11/14,657 (0.1)	1.37 (0.63, 2.99)
Meningitis	0/14,573 (0.0)	0/14,657 (0.0)	--
Other infection in clinical record	128/14,573 (0.9)	140/14,657 (1.0)	0.92 (0.72, 1.16)
Respiratory rate ≥ 60	435/14,573 (3.0)	448/14,657 (3.1)	0.99 (0.87, 1.12)
Stillbirth, death, or infection	2,300/14,658 (15.7)	2,319/14,757 (15.7)	1.00 (0.95, 1.06)

[1] Maternal infection includes occurrence of chorioamnionitis, endometritis, wound infection or other maternal infection (abdominopelvic abscess, mastitis/breast abscess or infection, pyelonephritis, pneumonia, and other bacterial infection) within 6 weeks.
[2] Participants with an adjudication completed indicating other bacterial infection documented in clinical record and no other study infections are classified as having other bacterial infection. Per this definition, participants with sepsis cannot be classified as having other bacterial infection.
[3] Neonatal infection is defined as diagnosis eye infection with swelling and drainage, skin infection with 10 or more pustules or bullae, omphalitis, urinary tract infection, pyelonephritis or kidney infection, pneumonia or lung infection, meningitis, other infection documented in clinical record, or respiratory rate $\geq$ 60 within 4 weeks for neonates without a sepsis diagnosis.
[4] Estimated relative risk and 95% confidence interval of each outcome are presented. Estimates were obtained by fitting a Poisson model to each outcome adjusting for site and treatment. Models for neonatal outcomes account for correlation among multiples assuming an exchangeable covariance structure. Models for skin infection, urinary tract infection and pneumonia had convergence issues, so models are fit without the adjustment for correlation among multiples. Models for chorioamnionitis, abdominopelvic abscess without sepsis, pneumonia without sepsis, pyelonephritis/kidney infection and meningitis did not converge.

Figure 1. Participant flow Diagram

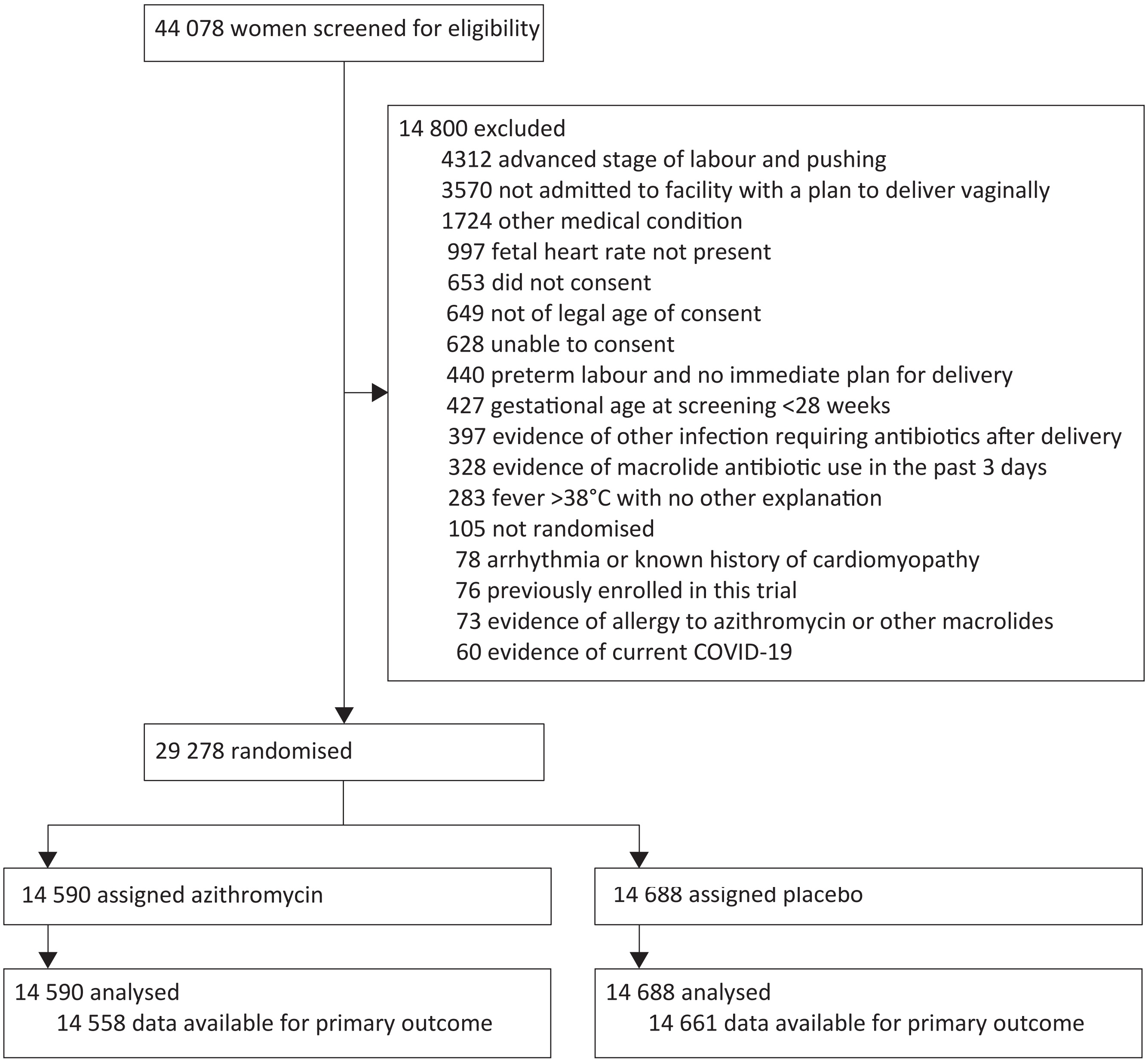

Figure 2. Any Maternal Infection Subgroup Analyses

Maternal Outcome Sub-Group	Azithromycin N=14,558	Placebo N=14,661	Int. P	RR (95% CI)
Any infection within 6 weeks	580/14,558 (4·0)	824/14,661 (5·6)		0·71 (0·64, 0·79)
High-risk cohort				
Yes	65/1,245 (5·2)	88/1,280 (6·9)	0·73	0·75 (0·54, 1·03)
No	515/13,312 (3·9)	736/13,381 (5·5)		0·71 (0·63, 0·79)
Geographic region				
Africa	108/5,777 (1·9)	222/5,794 (3·8)	0·0002	0·49 (0·39, 0·61)
Asia	458/7,989 (5·7)	582/8,066 (7·2)		0·79 (0·70, 0·90)
Prophylactic antibiotic use during labor				
Yes	149/2,042 (7·3)	205/2,038 (10·1)	0·81	0·73 (0·59, 0·90)
No	431/12,516 (3·4)	619/12,623 (4·9)		0·70 (0·62, 0·80)
Type of delivery				
Cesarean delivery	173/2,038 (8·5)	254/2,063 (12·3)	0·76	0·69 (0·57, 0·84)
Vaginal delivery	407/12,520 (3·3)	570/12,598 (4·5)		0·72 (0·63, 0·82)
Type of labor				
Spontaneous labor	387/11,909 (3·2)	577/11,938 (4·8)	0·13	0·67 (0·59, 0·76)
Induction	192/2,641 (7·3)	247/2,713 (9·1)		0·80 (0·66, 0·97)
Preterm				
Yes	71/1,834 (3·9)	106/1,893 (5·6)	0·68	0·67 (0·50, 0·90)
No	509/12,719 (4·0)	718/12,765 (5·6)		0·72 (0·64, 0·80)

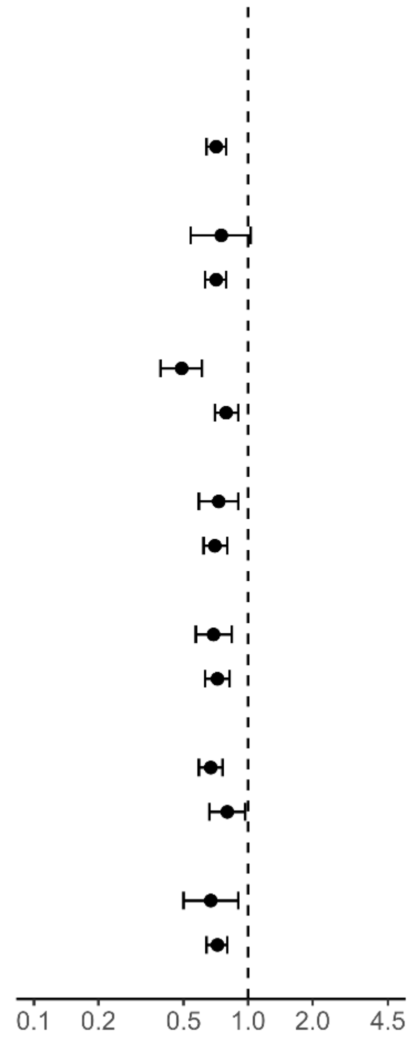

Forest plot displaying the estimated relative risk (and 95% confidence interval) of maternal sepsis and/or infection comparing mothers randomized to azithromycin versus placebo overall and within selected subgroups. Overall estimates were obtained by fitting a Poisson model adjusting for site and treatment arm. Subgroup estimates were obtained by fitting a Poisson model adjusting for site, treatment arm, subgroup, and the interaction of treatment arm and subgroup.

Figure 3. Maternal Sepsis Plus Another Infection Subgroup Analyses

Maternal Outcome Sub-Group	Azithromycin N=14,558	Placebo N=14,658	Int. P	RR (95% CI)
Sepsis plus infection within 6 weeks	150/14,558 (1·0)	261/14,658 (1·8)		0·58 (0·47, 0·71)
High-risk cohort				
Yes	16/1,245 (1·3)	20/1,279 (1·6)	0·34	0·79 (0·41, 1·52)
No	134/13,312 (1·0)	241/13,379 (1·8)		0·56 (0·45, 0·69)
Geographic region				
Africa	55/5,777 (1·0)	138/5,793 (2·4)	0·001	0·40 (0·29, 0·55)
Asia	94/7,989 (1·2)	122/8,064 (1·5)		0·78 (0·60, 1·02)
Prophylactic antibiotic use during labor				
Yes	28/2,042 (1·4)	51/2,038 (2·5)	0·84	0·56 (0·35, 0·88)
No	122/12,516 (1·0)	210/12,620 (1·7)		0·59 (0·47, 0·73)
Type of delivery				
Cesarean delivery	39/2,038 (1·9)	79/2,061 (3·8)	0·51	0·53 (0·36, 0·77)
Vaginal delivery	111/12,520 (0·9)	182/12,597 (1·4)		0·61 (0·48, 0·77)
Type of labor				
Spontaneous labor	102/11,909 (0·9)	191/11,936 (1·6)	0·22	0·54 (0·42, 0·68)
Induction	48/2,641 (1·8)	70/2,712 (2·6)		0·71 (0·49, 1·02)
Preterm				
Yes	24/1,834 (1·3)	46/1,893 (2·4)	0·71	0·53 (0·32, 0·87)
No	126/12,719 (1·0)	215/12,762 (1·7)		0·59 (0·47, 0·73)

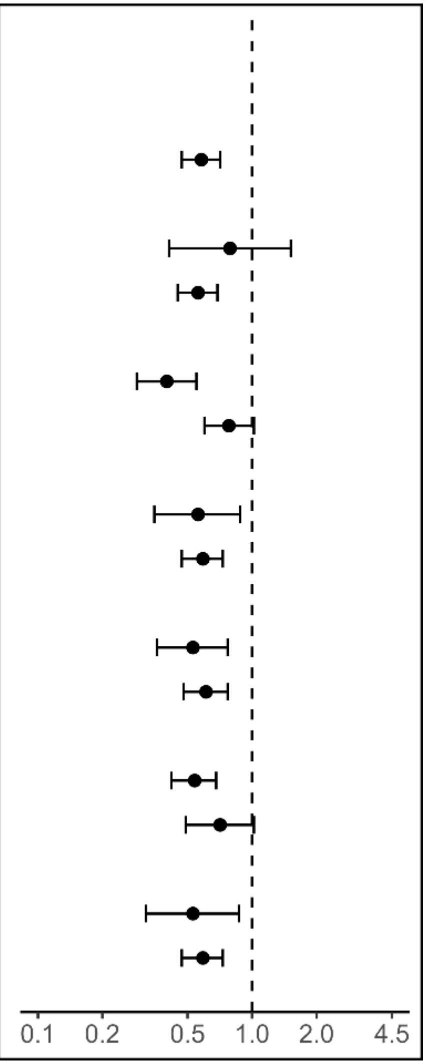

Forest plot displaying the estimated relative risk (and 95% confidence interval) of maternal sepsis with infection comparing mothers randomized to azithromycin versus placebo overall and within selected subgroups. Overall estimates were obtained by fitting a Poisson model adjusting for site and treatment arm. Subgroup estimates were obtained by fitting a Poisson model adjusting for site, treatment arm, subgroup, and the interaction of treatment arm and subgroup.

Figure 4. Maternal Sepsis Without Other Infection Subgroup Analyses

Maternal Outcome Sub-Group	Azithromycin N=14,558	Placebo N=14,658	Int. P	RR (95% CI)
Sepsis without infection within 6 weeks	69/14,558 (0·5)	76/14,658 (0·5)		0·91 (0·66, 1·26)
High-risk cohort				
Yes	7/1,245 (0·6)	16/1,279 (1·3)	0·09	0·45 (0·19, 1·10)
No	62/13,312 (0·5)	60/13,379 (0·4)		1·03 (0·73, 1·48)
Geographic region				
Africa	26/5,777 (0·5)	36/5,793 (0·6)	0·23	0·72 (0·44, 1·20)
Asia	43/7,989 (0·5)	40/8,064 (0·5)		1·08 (0·70, 1·67)
Prophylactic antibiotic use during labor				
Yes	13/2,042 (0·6)	7/2,038 (0·3)	0·12	1·79 (0·71, 4·49)
No	56/12,516 (0·4)	69/12,620 (0·5)		0·82 (0·58, 1·17)
Type of delivery				
Cesarean delivery	25/2,038 (1·2)	17/2,061 (0·8)	0·07	1·48 (0·80, 2·74)
Vaginal delivery	44/12,520 (0·4)	59/12,597 (0·5)		0·75 (0·51, 1·11)
Type of labor				
Spontaneous labor	44/11,909 (0·4)	57/11,936 (0·5)	0·18	0·77 (0·52, 1·15)
Induction	24/2,641 (0·9)	19/2,712 (0·7)		1·27 (0·70, 2·32)
Preterm				
Yes	15/1,834 (0·8)	13/1,893 (0·7)	0·41	1·21 (0·57, 2·54)
No	54/12,719 (0·4)	63/12,762 (0·5)		0·86 (0·59, 1·23)

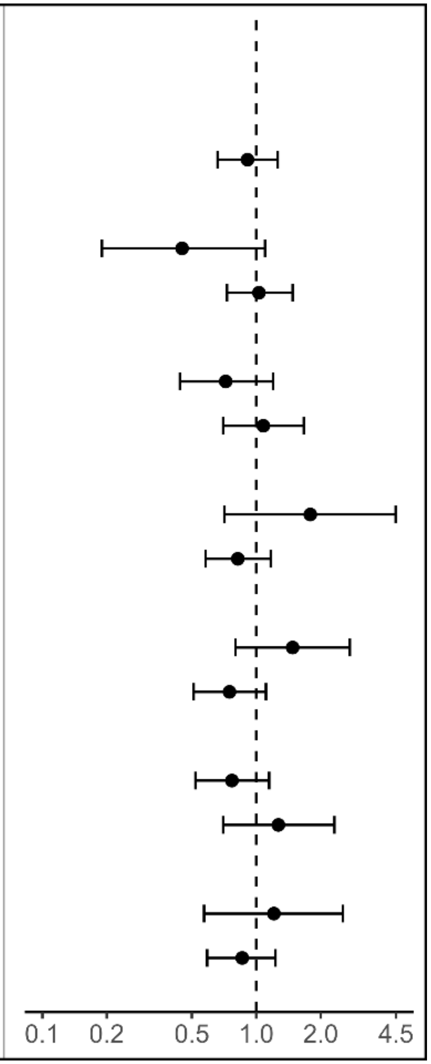

Forest plot displaying the estimated relative risk (and 95% confidence interval) of maternal sepsis without infection comparing mothers randomized to azithromycin versus placebo overall and within selected subgroups. Overall estimates were obtained by fitting a Poisson model adjusting for site and treatment arm. Subgroup estimates were obtained by fitting a Poisson model adjusting for site, treatment arm, subgroup, and the interaction of treatment arm and subgroup.

Supplementary materials Figure 1. Any Maternal Infection or Death Subgroup Analyses

Maternal Outcome Sub-Group	Azithromycin N=14,526	Placebo N=14,640	Int. P	RR (95% CI)
Death or any infection within 6 weeks	588/14,526 (4·0)	829/14,640 (5·7)		0·72 (0·64, 0·80)
High-risk cohort				
Yes	66/1,241 (5·3)	88/1,279 (6·9)	0·70	0·76 (0·55, 1·05)
No	522/13,285 (3·9)	741/13,361 (5·5)		0·71 (0·64, 0·79)
Geographic region				
Africa	110/5,772 (1·9)	226/5,792 (3·9)	0·0002	0·49 (0·39, 0·61)
Asia	464/7,962 (5·8)	583/8,047 (7·2)		0·80 (0·71, 0·91)
Prophylactic antibiotic use during labor				
Yes	151/2,033 (7·4)	205/2,035 (10·1)	0·74	0·74 (0·60, 0·91)
No	437/12,493 (3·5)	624/12,605 (5·0)		0·71 (0·63, 0·80)
Type of delivery				
Cesarean delivery	176/2,037 (8·6)	256/2,059 (12·4)	0·76	0·70 (0·58, 0·85)
Vaginal delivery	412/12,489 (3·3)	573/12,581 (4·6)		0·72 (0·64, 0·82)
Type of labor				
Spontaneous labor	394/11,883 (3·3)	582/11,923 (4·9)	0·14	0·68 (0·60, 0·77)
Induction	193/2,635 (7·3)	247/2,707 (9·1)		0·81 (0·67, 0·97)
Preterm				
Yes	73/1,829 (4·0)	107/1,893 (5·7)	0·75	0·68 (0·51, 0·92)
No	515/12,692 (4·1)	722/12,744 (5·7)		0·72 (0·64, 0·81)

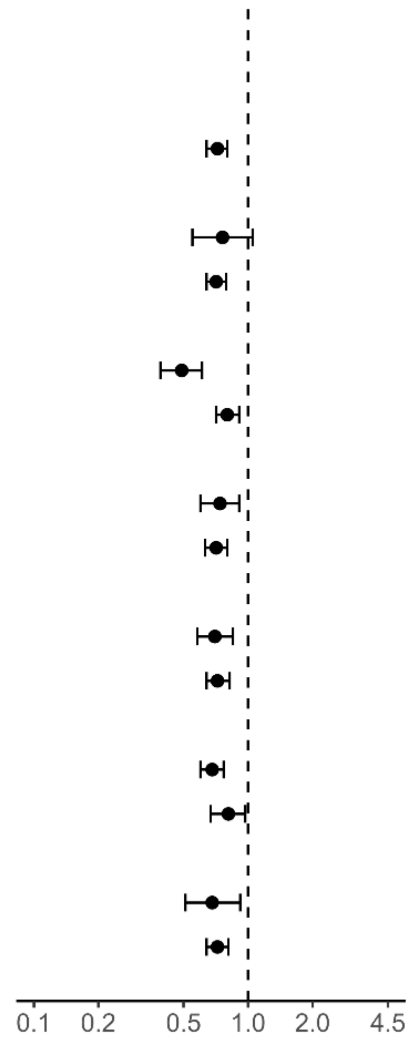

Forest plot displaying the estimated relative risk (and 95% confidence interval) of maternal death, sepsis, or infection comparing mothers randomized to azithromycin versus placebo overall and within selected subgroups. Overall estimates were obtained by fitting a Poisson model adjusting for site and treatment arm. Subgroup estimates were obtained by fitting a Poisson model adjusting for site, treatment arm, subgroup, and the interaction of treatment arm and subgroup.

Supplementary materials Figure 2. Maternal Infection without Sepsis Subgroup Analyses

Maternal Outcome Sub-Group	Azithromycin N=14,558	Placebo N=14,658	Int. P	RR (95% CI)
Infection without sepsis within 6 weeks	361/14,558 (2·5)	485/14,658 (3·3)		0·75 (0·66, 0·86)
High-risk cohort				
Yes	42/1,245 (3·4)	51/1,279 (4·0)	0·57	0·84 (0·56, 1·26)
No	319/13,312 (2·4)	434/13,379 (3·2)		0·74 (0·64, 0·86)
Geographic region				
Africa	27/5,777 (0·5)	48/5,794 (0·8)	0·21	0·56 (0·35, 0·90)
Asia	321/7,989 (4·0)	418/8,063 (5·2)		0·78 (0·67, 0·90)
Prophylactic antibiotic use during labor				
Yes	108/2,042 (5·3)	147/2,038 (7·2)	0·83	0·73 (0·57, 0·94)
No	253/12,516 (2·0)	338/12,620 (2·7)		0·76 (0·64, 0·89)
Type of delivery				
Cesarean delivery	109/2,038 (5·3)	157/2,062 (7·6)	0·48	0·70 (0·55, 0·89)
Vaginal delivery	252/12,520 (2·0)	328/12,596 (2·6)		0·78 (0·66, 0·91)
Type of labor				
Spontaneous labor	241/11,909 (2·0)	328/11,937 (2·7)	0·62	0·73 (0·62, 0·87)
Induction	120/2,641 (4·5)	157/2,711 (5·8)		0·79 (0·62, 1·00)
Preterm				
Yes	32/1,834 (1·7)	47/1,893 (2·5)	0·57	0·66 (0·42, 1·04)
No	329/12,719 (2·6)	438/12,762 (3·4)		0·76 (0·66, 0·88)

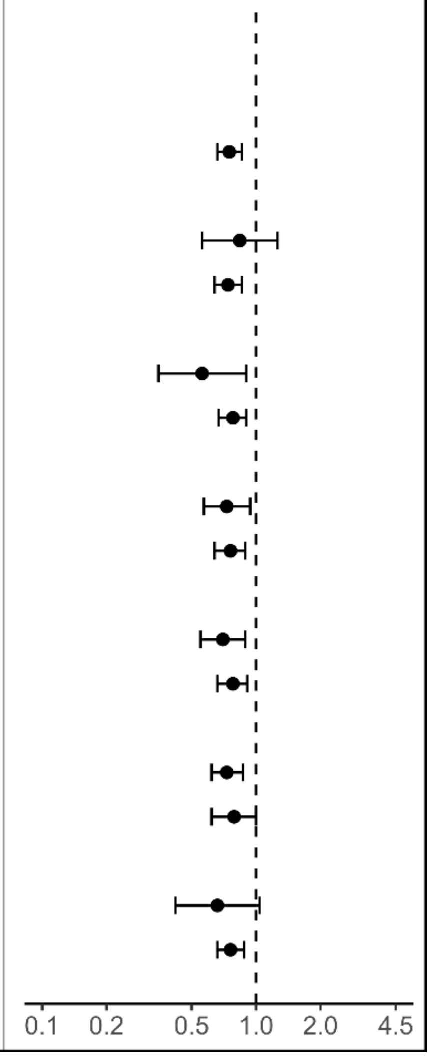

Forest plot displaying the estimated relative risk (and 95% confidence interval) of maternal infection without sepsis comparing mothers randomized to azithromycin versus placebo overall and within selected subgroups. Overall estimates were obtained by fitting a Poisson model adjusting for site and treatment arm. Subgroup estimates were obtained by fitting a Poisson model adjusting for site, treatment arm, subgroup, and the interaction of treatment arm and subgroup.

Supplementary materials Figure 3. Any Neonatal Infection Subgroup Analyses

Newborn Outcome Sub-Group	Azithromycin N=14,573	Placebo N=14,657	Int. P	RR (95% CI)
Any infection within 4 weeks	2,196/14,573 (15·1)	2,205/14,657 (15·0)		1·01 (0·96, 1·06)
High-risk cohort				
Yes	277/1,241 (22·3)	314/1,279 (24·6)	0·09	0·91 (0·79, 1·03)
No	1,918/13,331 (14·4)	1,891/13,378 (14·1)		1·02 (0·97, 1·08)
Geographic region				
Africa	474/5,790 (8·2)	492/5,796 (8·5)	0·57	0·98 (0·87, 1·10)
Asia	1,656/7,991 (20·7)	1,650/8,062 (20·5)		1·02 (0·96, 1·08)
Prophylactic antibiotic use during labor				
Yes	478/2,048 (23·3)	446/2,033 (21·9)	0·22	1·07 (0·96, 1·19)
No	1,718/12,525 (13·7)	1,759/12,624 (13·9)		0·99 (0·93, 1·05)
Type of delivery				
Cesarean delivery	410/2,037 (20·1)	420/2,058 (20·4)	0·98	1·01 (0·90, 1·13)
Vaginal delivery	1,786/12,536 (14·2)	1,785/12,599 (14·2)		1·01 (0·95, 1·07)
Type of labor				
Spontaneous labor	1,630/11,935 (13·7)	1,644/11,947 (13·8)	0·23	0·99 (0·93, 1·06)
Induction	566/2,630 (21·5)	559/2,700 (20·7)		1·07 (0·97, 1·18)
Preterm				
Yes	338/1,859 (18·2)	317/1,915 (16·6)	0·24	1·09 (0·95, 1·24)
No	1,858/12,708 (14·6)	1,888/12,739 (14·8)		0·99 (0·94, 1·05)

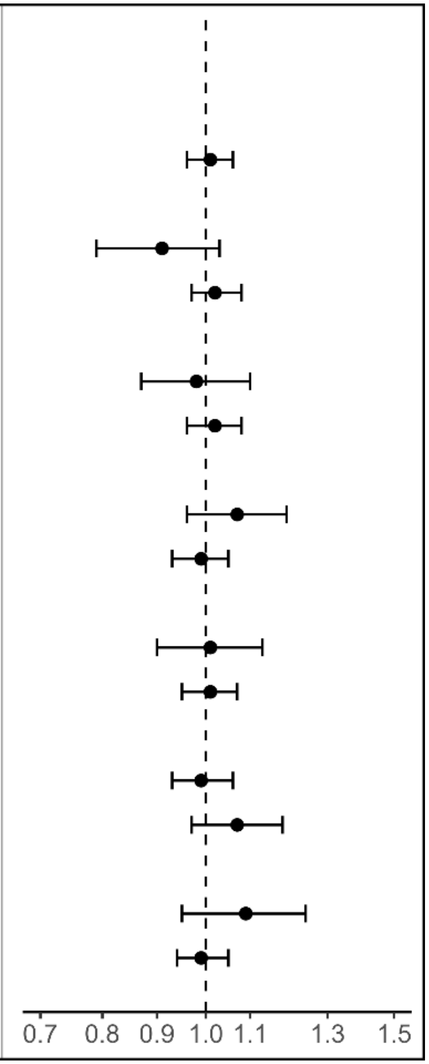

Forest plot displaying the estimated relative risk (and 95% confidence interval) of newborn sepsis and/or infection comparing newborns of mothers randomized to azithromycin versus placebo overall and within selected subgroups. Overall estimates were obtained by fitting a Poisson model adjusting for site and treatment arm. Subgroup estimates were obtained by fitting a Poisson model adjusting for site, treatment arm, subgroup, and the interaction of treatment arm and subgroup. Models for neonatal outcomes account for correlation among multiples assuming an exchangeable covariance structure.

Supplementary materials Figure 4. Neonatal Infection Without Sepsis Subgroup Analyses

Newborn Outcome Sub-Group	Azithromycin N=14,573	Placebo N=14,657	Int. P	RR (95% CI)
Infection without sepsis within 4 weeks	763/14,573 (5·2)	798/14,657 (5·4)		0·97 (0·88, 1·07)
High-risk cohort				
Yes	122/1,241 (9·8)	130/1,279 (10·2)	0·88	0·95 (0·77, 1·19)
No	640/13,331 (4·8)	668/13,378 (5·0)		0·97 (0·88, 1·08)
Geographic region				
Africa	149/5,790 (2·6)	165/5,796 (2·8)	0·55	0·92 (0·74, 1·14)
Asia	604/7,991 (7·6)	622/8,062 (7·7)		0·99 (0·89, 1·09)
Prophylactic antibiotic use during labor				
Yes	156/2,048 (7·6)	150/2,033 (7·4)	0·43	1·05 (0·85, 1·28)
No	607/12,525 (4·8)	648/12,624 (5·1)		0·95 (0·86, 1·06)
Type of delivery				
Cesarean delivery	130/2,037 (6·4)	130/2,058 (6·3)	0·41	1·06 (0·85, 1·32)
Vaginal delivery	633/12,536 (5·0)	668/12,599 (5·3)		0·96 (0·86, 1·06)
Type of labor				
Spontaneous labor	565/11,935 (4·7)	591/11,947 (4·9)	0·50	0·96 (0·86, 1·07)
Induction	198/2,630 (7·5)	207/2,700 (7·7)		1·03 (0·86, 1·22)
Preterm				
Yes	106/1,859 (5·7)	102/1,915 (5·3)	0·36	1·09 (0·84, 1·41)
No	657/12,708 (5·2)	696/12,739 (5·5)		0·96 (0·86, 1·06)

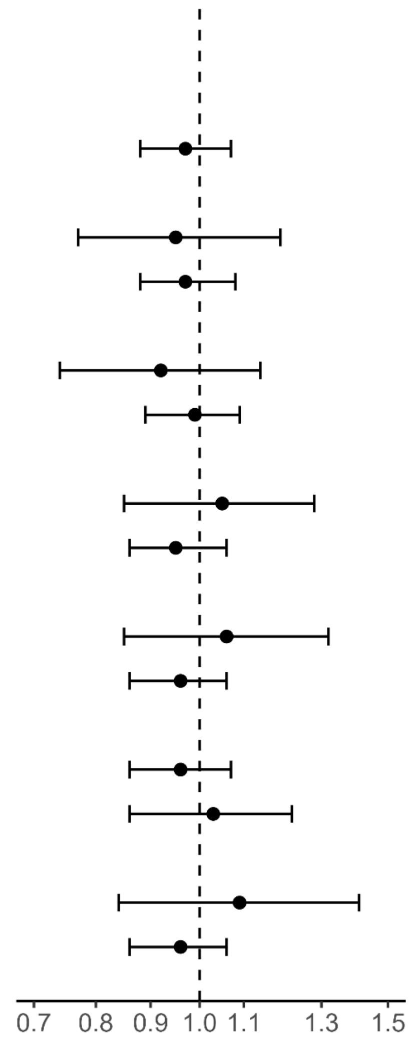

Forest plot displaying the estimated relative risk (and 95% confidence interval) of newborn infection without sepsis comparing newborns of mothers randomized to azithromycin versus placebo overall and within selected subgroups. Overall estimates were obtained by fitting a Poisson model adjusting for site and treatment arm. Subgroup estimates were obtained by fitting a Poisson model adjusting for site, treatment arm, subgroup, and the interaction of treatment arm and subgroup. Models for neonatal outcomes account for correlation among multiples assuming an exchangeable covariance structure.

Supplementary materials Figure 5. Stillbirth, Neonatal Death, and/or Any Infection Subgroup Analyses

Newborn Outcome Sub-Group	Azithromycin N=14,658	Placebo N=14,757	Int. P	RR (95% CI)
SB, ND or any infection within 4 weeks	2,300/14,658 (15·7)	2,319/14,757 (15·7)		1·00 (0·95, 1·06)
High-risk cohort				
Yes	289/1,252 (23·1)	324/1,289 (25·1)	0·15	0·92 (0·81, 1·04)
No	2,010/13,405 (15·0)	1,995/13,468 (14·8)		1·02 (0·96, 1·08)
Geographic region				
Africa	530/5,838 (9·1)	554/5,854 (9·5)	0·45	0·97 (0·86, 1·08)
Asia	1,703/8,028 (21·2)	1,698/8,102 (21·0)		1·02 (0·96, 1·08)
Prophylactic antibiotic use during labor				
Yes	490/2,057 (23·8)	463/2,046 (22·6)	0·25	1·06 (0·96, 1·18)
No	1,810/12,601 (14·4)	1,856/12,711 (14·6)		0·99 (0·93, 1·05)
Type of delivery				
Cesarean delivery	430/2,054 (20·9)	438/2,073 (21·1)	0·85	1·02 (0·91, 1·14)
Vaginal delivery	1,870/12,604 (14·8)	1,881/12,684 (14·8)		1·00 (0·95, 1·06)
Type of labor				
Spontaneous labor	1,711/12,004 (14·3)	1,734/12,025 (14·4)	0·19	0·99 (0·93, 1·05)
Induction	589/2,646 (22·3)	583/2,722 (21·4)		1·07 (0·97, 1·17)
Preterm				
Yes	369/1,883 (19·6)	350/1,943 (18·0)	0·23	1·08 (0·95, 1·23)
No	1,930/12,768 (15·1)	1,968/12,810 (15·4)		0·99 (0·94, 1·05)

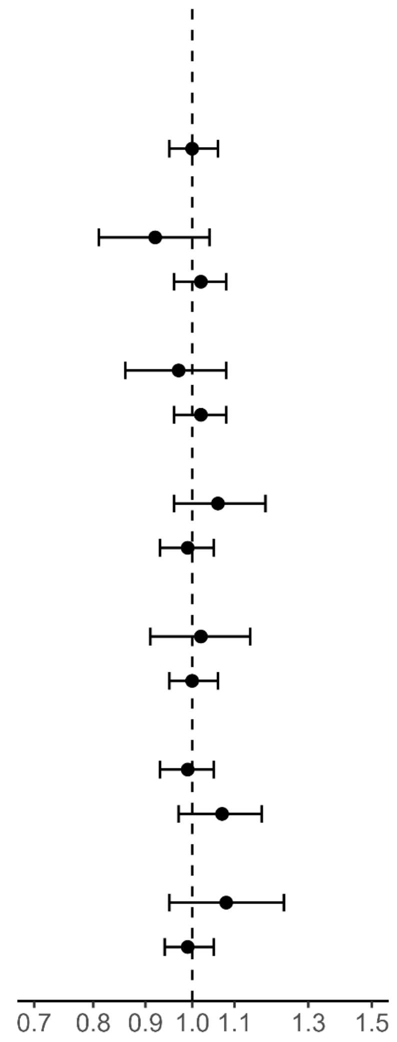

Forest plot displaying the estimated relative risk (and 95% confidence interval) of stillbirth (SB), neonatal death (ND), sepsis, or infection comparing newborns of mothers randomized to azithromycin versus placebo overall and within selected subgroups. Overall estimates were obtained by fitting a Poisson model adjusting for site and treatment arm. Subgroup estimates were obtained by fitting a Poisson model adjusting for site, treatment arm, subgroup, and the interaction of treatment arm and subgroup. Models for neonatal outcomes account for correlation among multiples assuming an exchangeable covariance structure.

A-PLUS Trial Group Members

First/Given Name	Family/Surname
Trecious	Mweemba
Ernest	Banda
Mwansa	Chimfwembe
Ruth	Nakazwe
Monica	Collins
Sixto	Leal
Akila	Subramaniam
Charitharth Vivek	Lal
Suchita	Parepalli
Anna	Aceituno
Jean	Kim
Kay	Jackson
Alexis	Williams
Marissa	Trotta
Menachem	Miodovnik
Jackie	Wallace
Gustave	Lomendje
Michel	Kalonji
Miyalu	Junior
Jackie	Patterson
Paulin	Takoy
Justin	Gado
Joel	Bossenya
Emmanuel	Kalombo
Charles	Kombi
Maynor	Manrique
Jamie	Westcott
Shahjahan	Siraj
Wazi	Sadeq-ur-Rahman
Lolit	Singh
Amita	Farzana
Farhana	Jahan
Zarin Tasmin	Maliha
Rumpa	Kairy
Christian	Chisolm
Robert A.	Sinkin

Manjunath S.	Somannavar
Kadappa	Beniwadi
Sheetal U.	Harkuni
Madiwalayya S.	Ganarchari
Umesh R.	Hundekar
Ashadevi	Patil
Ashwini	Bharamashetti
Netravathi	Alur
Surekha	Nyamagoud
Chandrashekhar	Kajagar
Sangappa M.	Dhaded
Ashalata A.	Mallapur
Ramesh	Pol
Geetanjali M.	Katageri
Umesh Y.	Ramadurg
Bhuvaneshwari C.	Yelamali
Aarti	Bhurle
Shailaja R.	Bidri
Sangamesh S.	Mathapati
Mallanagowda M.	Patil
Preeti G.	Patil
Hidayatullah R.	Bijapure
Chandrika R.	Doddihal
Muttu R.	Gudadinni
Smita O.	Bagali
Frances	Jaeger
Farnaz	Naqvi
Naija Karim	Ghanchi
Zaheer	Habib
Imran	Ahmed
Sana	Roujani
Seemab	Naqvi
Sayyeda	Reza
Haleema	Yasmin
Mashal	Khan
Mehmood	Shaikh
Hayat	Bozdar
Prabir	Dad
Kunal G.	Kurhe

Vaishali	Khedikar
Chaitali	Gedam
Savita	Bhargav
Samreen	Sadaf
Deepti	Shrivastava
Abhay	Gaidhane
Mugdha	Jungari
Manish	Jain
Manisha	Nasre
Sunanda	Shrikhande
Vijayshri	Deotale
Edwar A.	Liechty
Amos	Sagwe
Kevin	Otieno
Milsort	Kemboi
Anderson	Misati
Gabriel	Kigen
